# The Comparison of the Kidney Effects of Dipeptidyl Peptidase 4 Inhibitors and Glucagon-Like Peptide 1 Agonist-Administered Concomitant with Sodium-Glucose Cotransporter 2 Inhibitors in Japanese Patients with Type 2 Diabetes Mellitus and Chronic Kidney Disease

**DOI:** 10.1155/2021/6573369

**Published:** 2021-12-21

**Authors:** Kazuo Kobayashi, Masao Toyoda, Nobuo Hatori, Kazuyoshi Sato, Masaaki Miyakawa, Kouichi Tamura, Akira Kanamori

**Affiliations:** ^1^Committee of Hypertension and Kidney Disease, Kanagawa Physicians Association, Yokohama, Japan; ^2^Department of Medical Science and Cardiorenal Medicine, Yokohama City University Graduate School of Medicine, Yokohama, Japan; ^3^Department of Internal Medicine, Division of Nephrology, Endocrinology and Metabolism, Tokai University School of Medicine, Isehara, Japan

## Abstract

**Methods:**

We retrospectively constructed database of 763 Japanese patients with T2DM and CKD who received sSGLT2is for more than 1 year. Among these SGLT2i-treated patients, 338 were receiving concomitant DPP4i (DPP4i group), and 99 were receiving concomitant GLP1Ra (GLP1Ra group). The two groups were compared using the propensity score matching method.

**Results:**

In the matched model including 86 cases per group, the decrease in the logarithmic value of the ACR and rate of reduction in the estimated glomerular filtration rate (eGFR; mL/min/1.73 m^2^) of the GLP1Ra group showed no significant difference from those in the DPP4i group (−0.12 ± 0.48 vs. −0.13 ± 0.45 and −2.3 ± 18.5 vs. −6.2 ± 13.8, respectively, *P* = 0.10). However, the incidence of a >6.4% decrease in the eGFR was significantly lower in the GLP1Ra group than in the DPP4i group (35% vs. 52%, respectively, *P* = 0.03). The level of hemoglobin A_1c_ (mmol/mol) after SGLT2i treatment was significantly lower in the DPP4i group than in the GLP1Ra group in the matched model (58.3 ± 11.8 and 62.7 ± 14.8, respectively, *P* = 0.02).

**Conclusion:**

Among the SGLT2i-treated patients with T2DM and CKD, concomitant treatment with GLP1Ra has a marked improving effect on the change in the eGFR.

## 1. Introduction

Cardiovascular outcome trials (CVOTs) using empagliflozin (EMPA-REG OUTCOME trial) [1, 2], dapagliflozin (DECLARE-TIMI58) [3], and canagliflozin (CANVAS/CANVAS-R) [4] have demonstrated improvements in not only cardiovascular but also renal events. Our retrospective survey also revealed the improvement in the urine albumin-to-creatinine ratio (ACR) in Japanese patients with type 2 diabetes mellitus (T2DM) and chronic kidney disease (CKD) in clinical practice [5]. On our database of 763 SGLT2i-treated patients with T2DM and CKD, only 64 (8%) were treated with an SGLT2i alone; a majority needed concomitant hypoglycemic agents along with SGLT2i treatment. Among these agents, the incretin-related hypoglycemic agents; dipeptidyl peptidase 4 inhibitor (DPP4i) and glucagon-like peptide 1 agonist (GLP1Ra) were major concomitant medications, as they were administrated to more than 70% of SGLT2i-treated patients.

Both DPP4i and GLP1Ra are incretin-related drugs that reduce the plasma glucose level with an incretin effect, showing relatively similar results in CVOTs. DPP4i failed to show superiority compared with placebo for reducing the incidence of major adverse cardiovascular events (MACEs) [6–9], but some types of GLP1Ra did show superiority for reducing the incidence of MACEs and improving renal composite outcomes [10–13]. However, a head-to-head study comparing both of these agents for MACEs and renal outcomes has not been performed, and the differences in the renoprotective effects between these agents are poorly understood.

Accordingly, we performed a comparison to clarify the differences in renal effects between concomitant treatment with DPP4i and GLP1Ra in SGLT2i-treated patients.

## 2. Methods

### 2.1. Patients and Data Collection

This study was performed as a subanalysis of our previous reports [5]. In short, we collected the data of 763 T2DM participants with these inclusion criteria: (a) patients with CKD as defined by the clinical practice guidelines of the Kidney Disease Outcomes Quality Initiative, (b) patients who visited members of the Kanagawa Physicians Association from October to December 2018, and (c) patients who received first-time SGLT2i treatment for longer than 1 year.

Among these SGLT2i-treated patients, 338 had concomitant treatment with DPP4i (DPP4i group), and 99 had concomitant treatment with GLP1Ra (GLP1Ra group). These patients' clinical findings for gender, age, body weight (BW), diastolic and systolic blood pressure (DBP and SBP, respectively), hemoglobin A_1c_ (HbA_1c_) levels, serum creatinine levels, estimated glomerular filtration rate (eGFR), and ACR at the initiation of SGLT2i treatment and at the time of the survey were collected. The eGFR was calculated as follows: eGFR (mL/min/1.73 m^2^) = 194 × age − 0.287 × serum creatinine − 1.094 × (0.739 for women) [14].

Further analyses to compare these two groups were performed.

We conducted this study in compliance with the Declaration of Helsinki, and the special ethics committee of the Kanagawa Medical Association, Japan, approved this study (Krec304401.6 March 2018). Apart from the present survey, our group have already published a subanalysis of comparisons between the types of SGLT2is [15]. The participants in the present survey are the same as in the previous survey, and the similar methods of the statistical analysis using PS are used in the present survey.

### 2.2. Outcomes

The change in the eGFR (*Δ*eGFR) and the logarithmic value of ACR (*Δ*LNACR) were evaluated as the primary outcomes. Regarding the *Δ*eGFR, the incidence of a value more than the cut-off value, which was calculated by the receiver operating characteristic (ROC) curve of the *Δ*eGFR, was also evaluated. In addition, other clinical findings, including the HbA1c, BP, and BW, were also evaluated.

### 2.3. Statistical Analyses

The ROC curve was used to examine the overall prediction accuracy of the *Δ*eGFR and concomitant GLP1Ra treatment. The result was reported as the area under the curve (AUC). The cut-off value of the *Δ*eGFR for further analyses was determined from the results of the ROC analysis.

Statistical analyses were performed using propensity scores (PSs). We divided the SGLT2i-treated patients into two groups: the DPP4i group (*n* = 338) and the GLP1Ra group (*n* = 99). The PSs of patients in the GLP1Ra group were calculated by a logistic regression model with continuous variables of age, BW, body mass index (BMI), SBP, DBP, HbA_1c_, ACR, eGFR, and creatinine clearance (CCr) at baseline and categorical variables of gender, types of SGLT2is, use of concomitant BP-lowering agents, statins, and hypoglycemic agents other than DPP4i or GLP1Ra. The following algorithm was used for PS matching: 1 : 1 nearest neighbor matching with a caliper value of 0.05, equal to a width of one-quarter the standard deviation (SD) of PS, and without replacement [16]. We compared the *Δ*eGFR, *Δ*LNACR, and clinical background characteristics of the two groups using an unpaired *t*-test for the unmatched cohort model and paired *t*-test for the matched cohort model. For the categorical data, chi-square and McNemar's tests were used for the unmatched and matched cohort models, respectively. McNemar's test was used for the comparison of the incidence of cases whose *Δ*eGFR exceeded the cut-off value on a matched model.

## 3. Results

### 3.1. PS-Matched Cohort Model

A PS-matched model was constructed with 86 cases in each group. The clinical characteristics at baseline in the unmatched and matched models are given in Table 1. There were significant differences between the DPP4i group and GLP1Ra group in the age, BW, BMI, DBP, HbA1c, eGFR, CCr, ACR, and the use of tofogliflozin, metformin, and insulin (*P* < 0.001, <0.001, 0.03, 0.002, <0.001, 0.04, 0.001, 0.04, 0.02, 0.04, and 0.001, respectively).

There were no significant differences between the groups in the PS-matched model. The absolute standardized difference of $<1.96\times \sqrt{2/n}$ for measured covariates indicated that the balance between the groups was appropriate [17]. This borderline in the present matched cohort model (*n* = 86 per group) was 0.30 ($=1.96\times \sqrt{2/86}$), and all standardized differences in clinical characteristics were <0.30 in this matched cohort model.

The histograms of PS before and after matching are shown in Figure S1.

### 3.2. The Comparison of the Renal Composite Outcomes for the 86 Propensity-Matched Patients in Each Group

Clinical findings after SGLT2 inhibitor treatment in both models are shown in Table 2. In the matched cohort model including 86 cases in each group, the *Δ*LNACR and *Δ*eGFR of the patients in the GLP1Ra group showed no significant difference from those in the DPP4i group (−0.12 ± 0.48 vs. −0.13 ± 0.45 and −2.3 ± 18.5 vs. −6.2 ± 13.8, respectively, *P* = 0.10).

**Figure 1:**
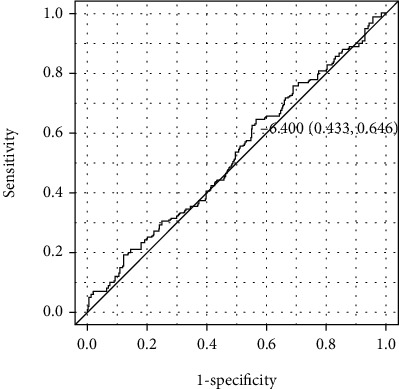
The receiver operating characteristic curve of the change in the eGFR and the concomitant use of GLP1Ra.

The ROC analysis (Figure 1) indicated that the estimated optimal cut-off value of the *Δ*eGFR for concomitant GLP1Ra therapy was -6.4%, with a sensitivity of 65%, specificity of 43%, and AUC of 0.53 (95% confidence interval (CI): 0.46, 0.59; *P* < 0.001). The incidence of an ΔeGFR > 6.4% was significantly lower in the GLP1Ra group than in the DPP4i group (35% vs. 52%, respectively, *P* = 0.03).

Regarding other clinical findings after SGLT2i treatment, the HbA1c level was significantly lower in the DPP4i group than in the GLP1Ra group in the matched model (*P* = 0.02). There were no significant differences in the BW, BMI, SBP, or DBP after SGLT2i treatment on the matched model.

## 4. Discussion

While incretin-related agents commonly decrease the plasma glucose level through targeting the incretin system [18] and stimulating the insulin secretion while inhibiting glucagon secretion when glucose levels are high, the results of CVOTs for these agents differ. The TECOS study using sitagliptin [6], EXAMINE study using alogliptin [7], SAVOR-TIMI53 study using saxagliptin [8], and CARMELINA study using linagliptin [9] showed noninferiority, but did not show the superiority with regard to reducing the incidence of MACEs. In contrast, the LEADER study using liraglutide [10], SUSTAIN-6 study using weekly injectional semaglutide [11], REWIND study using duraglutide [12], and Harmoney outcome trials using albiglutide [13] showed superiority with regard to reducing the incidence of MACEs compared to placebo. Both DPP4i and GLP1Ra showed mild improving effects on the exacerbation of the ACR [6, 8–11, 19], but neither showed superiority compared to placebo with regard to events related to the *Δ*eGFR or the induction to kidney replacement therapy. Regarding metabolic factors, GLP1Ra treatment reduced the BW and BP and improved the lipid profile [10–13], findings not seen in CVOTs using DPP4i. Based on these previous findings, in clinical practice, it is important to understand the difference among incretin-related agents and determine their proper use.

The primary outcomes of this subanalysis were the *Δ*eGFR and *Δ*LNACR, and we noted no significant difference in these values between the DPP4 group and GLP1Ra group. However, the sample size of the patients in the GLP1Ra group was small, which is a limitation of this subanalysis. Regarding the *Δ*eGFR, the 95% CI was -8.5% to 0.8%, so a study with a larger sample size may show a significant difference in the *Δ*eGFR between the groups. CVOTs involving both DPP4i and GLP1Ra demonstrated their mild benefits in ameliorating the exacerbation of the ACR, but they showed no marked efficacy on the *Δ*eGFR or induction of kidney replacement therapy. Furthermore, sitagliptin in the TECOS study significantly reduced the eGFR compared to placebo, our results of this subanalysis that DPP4i may have some disadvantage in *Δ*eGFR compared to GLP1Ra were not contradictory to these facts.

In the present study, the cut-off value of -6.4% for the *Δ*eGFR was calculated using an ROC analysis, and the difference in the incidence of an ΔeGFR > −6.4% was determined. The National Kidney Foundation and US Food and Drug Administration concluded that a 30% to 40% reduction in the eGFR over 2 to 3 years may be an acceptable surrogate endpoint for clinical trials on CKD patients [20]. The Japanese Society of Nephrology also discussed the renal surrogate endpoint and stated that a 30% to 40% reduction in the eGFR over 2 or 3 years is acceptable as a surrogate endpoint for Japanese CKD patients [21]. The meaning of such a small cut-off value of -6.4% is unclear, but the advantage in not the change in ACR but the change in eGFR might play some important role for not only renal but also cardiovascular protective effect by GLP1Ra.

In the SUSTAIN-2 study, semaglutide decreased the HbA1c and BW values to a greater degree than sitagliptin [22], however, in the present subanalysis, the greater decrease in the HbA1c value after SGLT2i treatment were observed In DPP4i group than in GLP1Ra group. Because the present subanalysis evaluated the influence of DPP4i or GLP1Ra as the concomitant partner of SGLT2i treatment, our present findings suggest that DPP4i may be a better partner than GLP1Ra for SGLT2i with regard to controlling the plasma glucose level.

Several potential reasons for the greater decrease in HbA1c with concomitant DPP4i versus GLP1Ra have been proposed. Kim et al. reported that the HbA1c-lowering efficacy of GLP1Ra was greater in studies with a mean baseline BMI < 30 kg/m^2^ than in those with a BMI ≥ 30 kg/m^2^ [23]. The BMI of the patients in the matched model of the present study was 28.9 kg/m^2^, whereas it was 32.5 kg/m^2^ in the SUSTAIN-2 study; these differences may influence the HbA1c-lowering effect of GLP1Ra. GLP1Ra lowers not only the HbA1c level but also the BW, and this BW-lowering effect contributes in part to the HbA1c-lowering effect. In the matched model of the present study, the *Δ*BW was almost the same between the groups (−3.7 ± 4.7 kg in the DPP4i group and −3.6 ± 5.7 kg in the GLP1Ra group). These findings may be involved in the decrease in HbA1c in the GLP1Ra group being smaller than expected. Furthermore, adherence to these agents may influence the HbA1c-lowering effect, as GLP1Ra is an injectional medication, whereas DPP4i is oral. However, the detailed reason is unclear at present, and further studies including an examination of the biological mechanism will be needed in the future.

Although CVOTs using DPP4is did not show the superiority of these agents to the placebo for reducing the incidence of MACEs, the use of DPP4is may be recommended for the management of DM patients, as better control of plasma glucose is achieved when SGLT2i-treated patients are administered DPP4is than GLP1Ras.

### 4.1. Study Limitations

Several limitations associated with the present subanalysis warrant mention. First, we compared these two agents as concomitant agents with SGLT2i. Therefore, our results do not necessarily reflect the accurate influence of each incretin-related agent. Second, this was a retrospective, observational study. This survey included only patients who could continue SGLT2i treatment and did not include those who stopped SGLT2i treatment or initiated renal replacement therapy during treatment. Therefore, the *Δ*eGFR and *Δ*ACR values may not have been accurate. We also could not evaluate the patients who stopped SGLT2i due to the adverse events (AEs). Mirabelli et al. reported the efficacy and the AEs of the long use of SGLT2i [24] and GLP1Ra in clinical practice retrospectively. Among 408 patients with T2DM, 66 patients stopped SGLT2i because of chronic or recurring genital infections [24]. They also reported that 13 out of 126 participants with T2DM discontinued dulaglutide due to moderate-severe gastrointestinal AEs [25]. In the present survey, we showed the superiority of the concomitant use of GLP1Ra during the SGLT2i treatment compared with the concomitant use of DPP4i, especially on the change in eGFR; however, the use of GLP1Ra and SGLT2i may increase the frequency of AEs or the deterioration of eGFR. Third, although PS methods can be advantageous compared with conventional statistical analyses for adjusting for confounders, 75% of the patients with concomitant DPP4i treatment were not selected in our PS-matched model.

## 5. Conclusion

Among the SGLT2i-treated patients with T2DM and CKD, concomitant treatment with GLP1Ra has a marked improving effect on the change in the eGFR.

## Figures and Tables

**Table 1:** Baseline characteristics before and after propensity score matching.

	Unmatched cohort (*n* = 437)			Matched cohort (*n* = 168)			
	DPP4i (*n* = 338)	GLP1Ra (*n* = 99)	*P* value	DPP4i (*n* = 86)	GLP1Ra (*n* = 86)	*P* value	Standardized difference
Age (years)	62.5 ± 11.5	56.6 ± 11.0	<0.001	56.1 ± 12.1	57.1 ± 11.0	0.55	0.09
Gender (male)	220 (65%)	65 (66%)	0.92∗	58 (67%)	58 (67%)	1.0☨	0.02
BW (kg)	76.8 ± 15.6	84.5 ± 17.4	<0.001	84.6 ± 16.7	84.2 ± 17.4	0.86	0.02
BMI (kg/m^2^)	27.4 ± 4.5	29.0 ± 5.7	0.01	28.9 ± 4.7	28.9 ± 5.8	0.99	<0.01
SBP (mmHg)	134.0 ± 16.5	136.5 ± 18.6	0.19	134.5 ± 16.9	135.6 ± 19.0	0.65	0.06
DBP (mmHg)	76.1 ± 11.8	80.4 ± 12.6	0.002	80.4 ± 12.3	80.1 ± 12.9	0.84	0.02
HbA1c (mmol/mol (%))	62.9 ± 14.0 (7.9 ± 1.3)	73.5 ± 15.2 (8.9 ± 1.4)	<0.001	72.0 ± 18.3 (8.7 ± 1.7)	72.3 ± 15.3 (8.8 ± 1.4)	0.88	0.02
eGFR (mL/min/1.73 m^2^)	77.3 ± 21.0	82.4 ± 24.2	0.04	82.0 ± 22.0	82.8 ± 25.8	0.91	0.03
CCr (mL/min)	113.4 ± 47.4	131.1 ± 52.2	0.001	135.5 ± 56.7	133.4 ± 54.1	0.81	0.04
ACR (*μ*g/gCr)	12.7 [6.3, 93.1]	18.0 [18.0, 145.1]	0.04	39.6 [13.0, 140.5]	41.6 [15.1, 145.7]	0.23	
LNACR	1.58 ± 0.64	1.74 ± 0.68	0.04	1.64 ± 0.67	1.72 ± 0.71	0.46	0.11
Duration of treatment (months)	32.9 ± 10.7	33.8 ± 10.8	0.50	31.8 ± 10.7	33.3 ± 10.5	0.36	0.14
Types of SGLT2 inhibitor							
Ipragliflozin	77 (23%)	21 (21%)	0.74^∗^	19 (22%)	21 (24%)	0.47^☨^	0.15
Dapagliflozin	57 (17%)	14 (14%)	0.52^∗^	11 (13%)	11 (13%)	0.21^☨^	0.23
Tofogliflozin	33 (10%)	18 (18%)	0.02^∗^	12 (14%)	14 (16%)	1.0^☨^	0.03
Luseogliflozin	29 (9%)	8 (8%)	0.88^∗^	8 (9%)	8 (9%)	0.61^☨^	0.12
Canagliflozin	45 (13%)	10 (10%)	0.40^∗^	11 (13%)	9 (11%)	0.52^☨^	0.14
Empagliflozin	51 (15%)	9 (9%)	0.13^∗^	7 (8%)	8 (9%)	1.0^☨^	0.0
SGLT2i changed during treatment	46 (14%)	19 (19%)	0.17^∗^	18 (21%)	15 (17%)	0.47^☨^	0.15
Concomitant treatment (at survey)							
Metformin	212 (63%)	73 (75%)	0.04^∗^	61 (71%)	62 (72%)	0.46^☨^	0.14
SU	122 (36%)	36 (36%)	0.96^∗^	29 (34%)	32 (37%)	0.87^☨^	0.05
Insulin	84 (25%)	42 (42%)	0.001^∗^	31 (36%)	33 (38%)	0.87^☨^	0.05
Pioglitazone	78 (23%)	15 (15%)	0.09^∗^	14 (16%)	14 (16%)	0.66^☨^	0.10
RAS inhibitors	172 (51%)	59 (60%)	0.13^∗^	47 (55%)	49 (57%)	1.0^☨^	0.02
Ca channel blocker	157 (46%)	39 (39%)	0.21^∗^	32 (37%)	34 (40%)	0.75^☨^	0.07
*Β* blocker	44 (13%)	15 (15%)	0.59^∗^	13 (15%)	14 (16%)	1.0^☨^	0.0
Statins	212 (63%)	62 (63%)	0.99^∗^	51 (59%)	54 (63%)	1.0^☨^	0.02

Values are shown as the mean ± SD or *n*/total *n* (%). *P* values by an unpaired *t*-test or ^∗^chi-square test with the unmatched cohort model and paired *t*-test and ^☨^McNemar's test with the matched cohort model. ^※^95% confidence interval of the logarithmic value of the odds ratio calculated by the Mantel-Haenszel method. ^¶^SGLT2 inhibitor changed during the study period. BW: body weight; BMI: body mass index; CCr: creatinine clearance; DPP4: dipeptidyl peptidase-4; eGFR: estimated glomerular filtration; GLP1RA: glucagon-like peptide 1 receptor agonist; LNACR: logarithmic value of albumin-to- creatinine ratio; MAP: mean arterial pressure; RAS: renin-angiotensin system inhibitor; SGLT2: sodium-glucose cotransporter.

**Table 2:** Clinical findings after SGLT2 inhibitor treatment in both models.

	Unmatched cohort (*n* = 437)			Matched cohort (*n* = 172)		
	DPP4i (*n* = 338)	GLP1Ra (*n* = 99)	*P* value	DPP4i (*n* = 86)	GLP1Ra (*n* = 86)	*P* value
(a) Primary outcomes of this subanalysis						
eGFR (mL/min/1.73 m^2^)	73.7 ± 21.7	75.1 ± 21.8	0.44	75.5 ± 19.6	79.8 ± 29.6	0.29
CCr (mL/min)	100.3 ± 41.8	125.8 ± 59.2	<0.001	119.8 ± 47.4	125.7 ± 61.7	0.49
*Δ*eGFR (%)	−5.2 ± 11.7	−2.6 ± 17.5	0.08	−6.2 ± 13.8	−2.3 ± 18.5	0.10
Annual *Δ*eGFR (mL/min/1.73 m^2^/year)	−2.0 ± 6.5	−1.4 ± 0.73	0.41	−2.2 ± 7.3	−1.4 ± 7.7	0.37
ACR	25.1 [10.4, 73.9]	34.9 [11.4, 125.4]	0.14	27.6 [9.7, 100.6]	31.2 [11.0, 125.6]	0.047
LNACR	1.48 ± 0.67	1.62 ± 0.70	0.08	1.52 ± 0.70	1.60 ± 0.74	0.45
*Δ*LNACR	−0.10 ± 0.46	−0.12 ± 0.47	0.66	−0.13 ± 0.05	−0.12 ± 0.05	0.90

(b) Other clinical findings after SGLT2i treatment						
BW (kg)	74.8 ± 16.0	77.5 ± 15.2	0.05	80.9 ± 15.9	80.6 ± 17.4	0.89
BMI (kg/m^2^)	26.5 ± 5.0	27.7 ± 6.5	0.08	27.8 ± 5.0	27.8 ± 6.7	0.93
SBP (mmHg)	129.1 ± 13.5/75.4 ± 10.4	129.0 ± 15.7/76.8 ± 11.4	0.93/0.14	126.7 ± 16.0	127.8 ± 15.7	0.66
DBP (mmHg)	93.3 ± 9.7	94.2 ± 11.3	0.33	75.4 ± 12.2	76.3 ± 11.8	0.62
HbA1c (mmol/mol (%))	55.7 ± 11.0 (7.2 ± 1.0)	57.0 ± 12.0 (7.4 ± 1.1)	0.18	58.3 ± 11.8 (7.5 ± 1.1)	62.7 ± 14.8 (7.9 ± 1.4)	0.02

Abbreviations are described in Table 1.

## Data Availability

The data are stored in the repository of The Kanagawa Physicians Association. The datasets used and analyzed during the current study are available from the corresponding author on reasonable request.

## Supplementary Materials

Supplementary Materials Supplementary Figure S1: the histograms of PS before and after matching.
