# Supplementary material for: The Comparison of the Kidney Effects of Dipeptidyl Peptidase 4 Inhibitors and Glucagon-Like Peptide 1 Agonist-Administered Concomitant with Sodium-Glucose Cotransporter 2 Inhibitors in Japanese Patients with Type 2 Diabetes Mellitus and Chronic Kidney Disease
Source: J Diabetes Res. 2021 Dec 21;2021:6573369. doi: 10.1155/2021/6573369 (PMC8749372; doi:10.1155/2021/6573369)

FIGURE S1 **Histogram of propensity score before and after matching**

a) Before propensity score matching

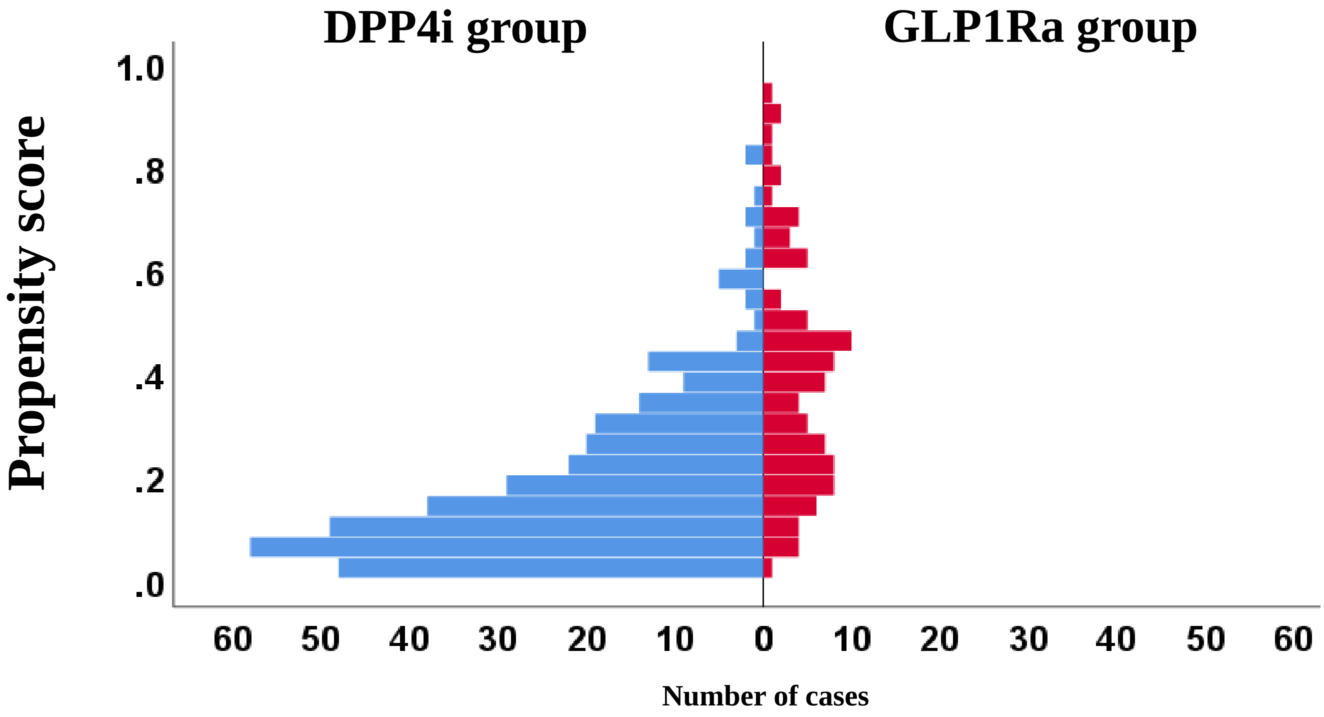

b) After propensity score matching

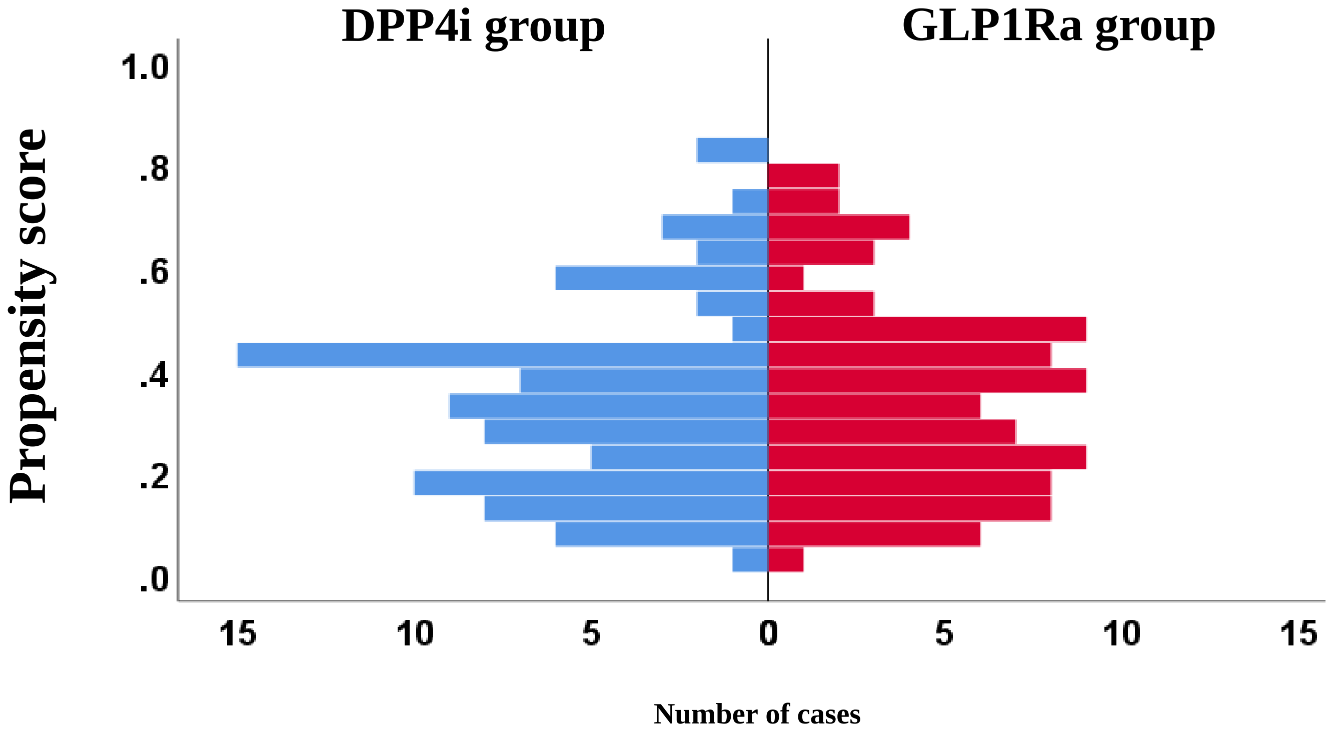

Supplement: Supplementary Materials — Supplementary Figure S1: the histograms of PS before and after matching. [file 6573369.f1.pdf]
